# plotnineSeqSuite: a Python package for visualizing sequence data using ggplot2 style

**DOI:** 10.1186/s12864-023-09677-8

**Published:** 2023-10-03

**Authors:** Tianze Cao, Qian Li, Yuexia Huang, Anshui Li

**Affiliations:** 1 School of Mathematics, Hangzhou Normal University, Hangzhou, Zhejiang Province China; 2 Department of Rehabilitation, Tongji Hospital, Tongji Medical College, Huazhong University of Science and Technology, Wuhan, Hubei Province China; 3 Department of Statistics, Shaoxing University, Shaoxing, Zhejiang Province China

**Keywords:** ggplot2, plotnine, Bioinformatics tool, Sequence logo, Multiple sequence alignment

## Abstract

**Background:**

The visual sequence logo has been a hot area in the development of bioinformatics tools. ggseqlogo written in R language has been the most popular API since it was published. With the popularity of artificial intelligence and deep learning, Python is currently the most popular programming language. The programming language used by bioinformaticians began to shift to Python. Providing APIs in Python that are similar to those in R can reduce the learning cost of relearning a programming language. And compared to ggplot2 in R, drawing framework is not as easy to use in Python. The appearance of plotnine (ggplot2 in Python version) makes it possible to unify the programming methods of bioinformatics visualization tools between R and Python.

**Results:**

Here, we introduce plotnineSeqSuite, a new plotnine-based Python package provides a ggseqlogo-like API for programmatic drawing of sequence logos, sequence alignment diagrams and sequence histograms. To be more precise, it supports custom letters, color themes, and fonts. Moreover, the class for drawing layers is based on object-oriented design so that users can easily encapsulate and extend it.

**Conclusions:**

plotnineSeqSuite is the first ggplot2-style package to implement visualization of sequence -related graphs in Python. It enhances the uniformity of programmatic plotting between R and Python. Compared with tools appeared already, the categories supported by plotnineSeqSuite are much more complete. The source code of plotnineSeqSuite can be obtained on GitHub (https://github.com/caotianze/plotnineseqsuite) and PyPI (https://pypi.org/project/plotnineseqsuite), and the documentation homepage is freely available on GitHub at (https://caotianze.github.io/plotnineseqsuite/).

**Supplementary Information:**

The online version contains supplementary material available at 10.1186/s12864-023-09677-8.

## Background

The sequence logo is a graphical representation of the results of multiple sequence alignments [1]. The abscissa of the sequence logo diagram represents the position of the aligned nucleic acid (or amino acid) and the letters representing the nucleic acid (or amino acid) are drawn closely stacked at each position. The height of each letter reflects the frequency of the nucleic acid (or amino acid) at the corresponding position. The stacking order of the letters is determined by the height of the letters. The tallest letter is stacked at the top of each position, and the shortest letter is stacked at the bottom. According to the calculation method of letter height, sequence logos is usually divided into two types: (1) The first type is called probability logo. Its ordinate ranges from 0 to 1. The height of the letter is equal to the frequency of occurrence of the nucleic acid (or amino acid) at the current position. The sum of the heights of the letters at each position is exactly 1. (2) The other one is called information logo, which can be used to display consensus sequence like probability logo but the calculation formula of its letter height is complex [see Additional file 1]. Furthermore, it can be used to represent protein-binding sites in deoxyribonucleic acid(DNA) or functional units in proteins [1].

Although there are many applications [2–25] that support drawing sequence logos, ggseqlogo [26], written in R language, has been the most interesting API since it was published. The reasons why it is so popular can be summarized into two points: (1) ggseqlogo provides an easy-to-use API so that programmers can easily create the sequence logos they need. (2) ggseqlogo is implemented based on ggplot2 [27] which provides powerful and easy-to-use APIs. In other words, programmers can easily use ggplot2 for secondary development of ggseqlgo.

Now, due to the rise of artificial intelligence and deep learning, Python has become the most popular programming language. The programming language used by bioinformaticians to develop bioinformatics tools began to change from Perl [28–31] and R [32, 33] to Python [34]. Since there was no one before API of Python provides an API similar to ggplot2, so there is no API of Python that implements API of ggseqlgo. With the release of the latest version of plotnine, it is possible to provide an API like ggseqlgo in Python. The latest version of plotnine almost implements API of ggplot2. It allows bioinformaticians to draw graphs in Python using almost the same API as ggplot2. Here we introduce a new Python package called plotnineSeqSuite, which can use almost the same code as ggseqlogo to draw sequence logo. Users can easily draw the same diagrams as R on the Python side with this package. In addition, plotnineSeqSuite can visualize sequence alignment diagrams and sequence histogram based on ggplot2 style.

### Implementation

plotnineSeqSuite development.

**Fig. 1:**
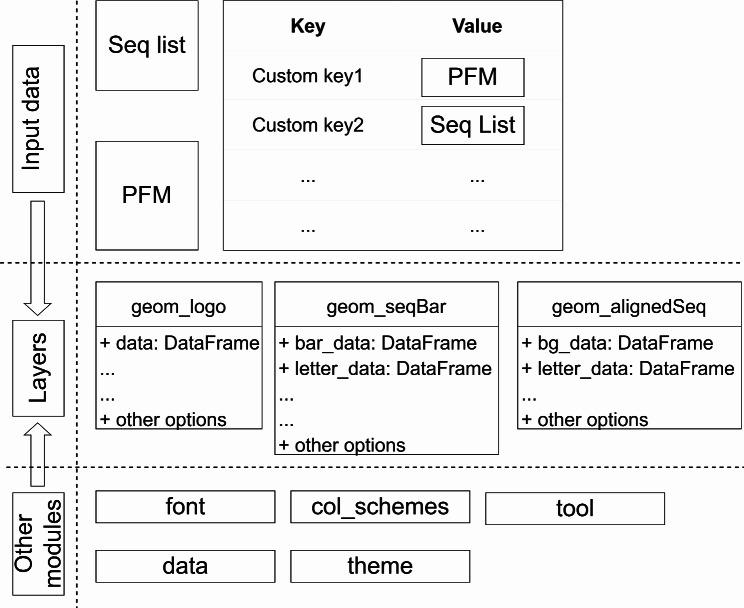
plotnineSeqSuite architecture overview

plotnineSeqSuite is written based on Python which depends on 3 Python packages: plotnine [36], pandas [37]and NumPy [38]. plotnine is the Python implementation of ggplot2, and plotnineSeqSuite uses the layer class and other auxiliary classes. pandas provides the Python version of DataFrame since the data source of plotnine drawing must be DataFrame. NumPy provides the function of mathematical matrix operation, and plotnineSeqSuite needs to calculate based on matrix in the process of processing data, which must be implemented with NumPy. The API provided by plotnineSeqSuite is distributed in 2 sub-packages and 6 modules (Fig. 1), the details of which are described below.

The sub-package data predefines 3 Python dictionaries, which preset nucleic acid and amino acid data for trial use of this software package. pfms_dna and seqs_dna define some transcription factors, and these data come from JASPAR [39]. The keys of its dictionary represents the JASPAR ID. seqs_aa defines some kinase-substrate phosphorylation sites, and these data come from the work of Wagih et al [40]. The keys of its dictionary represent the kinases associated with the phosphosites.

The sub-package font defines 15 built-in fonts and functions to obtain font data. The function list_fonts() is used to view the names of all fonts. The function get_font() can obtain font coordinate data based on the font name.

The Python classes geom_alignedSeq, geom_seqBar and geom_logo are defined in the modules align.py, bar.py and logo.py. The DataFrame used for plotnine drawing is predefined in these classes, such as the class property geom_logo.data. In order to meet the demonstration needs of these graphs, plotnineSeqSuite needs to modify the default coordinate axis information of the graph. plotnineSeqSuite also predefines some class properties to store the above information, such as class property geom_logo.xlab and geom_logo.scale_x_continuous. Users can use ggplot() to add these classes to get sequence logo, sequence alignment diagram and sequence histogram.

The constants and functions needed for color schemes are defined in the module col_schemes.py. The constant col_schemes gives the names of all the predefined color schemes. The function get_col_scheme() can get the specified color scheme based on the name of the color scheme. If the user is not satisfied with the color scheme provided by default, one can also use the function make_col_scheme() to customize the color scheme.

Moreover, the module theme.py defines a function theme_seq(), which provides a theme with a plain white background. The module tool.py provides a data processing auxiliary function extract(), which will be used to extract fragmented sequences from full-length sequences.

### DataFrame in plotnineSeqSuite

DataFrame is the data source for ggplot2 to draw graphics, which is equivalent specifically to pandas.DataFrame in Python. The types of geom_alignedSeq.bg_data(Table 1), geom_alignedSeq.letter_data(Table S1 [see Additional file 2]), geom_seqBar.bar_data(Table S2 [see Additional file 2]), geom_seqBar.letter_data(Table S3 [see Additional file 2]), geom_logo.data(Table S4 [see Additional file 2]) are DataFrame. They are the data sources for drawing their own graphics. Users can adjust the graphics by changing the data in the corresponding DataFrame, and even realize secondary development through object-oriented inheritance and encapsulation.

**Table 1:** Columns of geom_alignedSeq.bg_data

Number	Name	Example	Function
1	letter	T	The column is used to identify which letter the row is used to draw.
2	position	1	The column is used to identify at which position the data is used to plot the aligned sequences.
3	y_index	1	The column is used to identify index of y-axis at current aligned position.
4	x	1	The column is used to map x in geom_tile().
5	y	0.5	The column is used to map y in geom_tile().
6	width	1	The column is used to map width in geom_tile().
7	height	1	The column is used to map height in geom_tile().
8	seq_group	1	The column is used in facet_wrap().When the type of input data is dict, the value is the key value of dict. In other cases, the default is 1.
9	col	#D62839	When the color scheme is discrete, this column is used to specify the corresponding color. The column only exists when the parameter scheme_applied is ‘BACKGROUND’ when the class geom_alignedSeq is constructed.
10	group	T	The column is used to map fill in geom_tile().The column only exists when the parameter scheme_applied is ‘BACKGROUND’ when the class geom_alignedSeq is constructed.

## Results

### Input data

plotnineSeqSuite accepts three different types of input formats: list, NumPy.ndarray, and dict. Items of the list must be aligned sequences, while that of the NumPy.ndarray must be a position frequency matrix (PFM) which indicates how often individual characters appear at the specified position. The rows of the PFM are the letters and the columns of the PFM are the positions. Values of the dict are list or NumPy.ndarray described above and keys are identifiers that will be used as the facet titles (Fig. 1).

### Color schemes

**Fig. 2:**
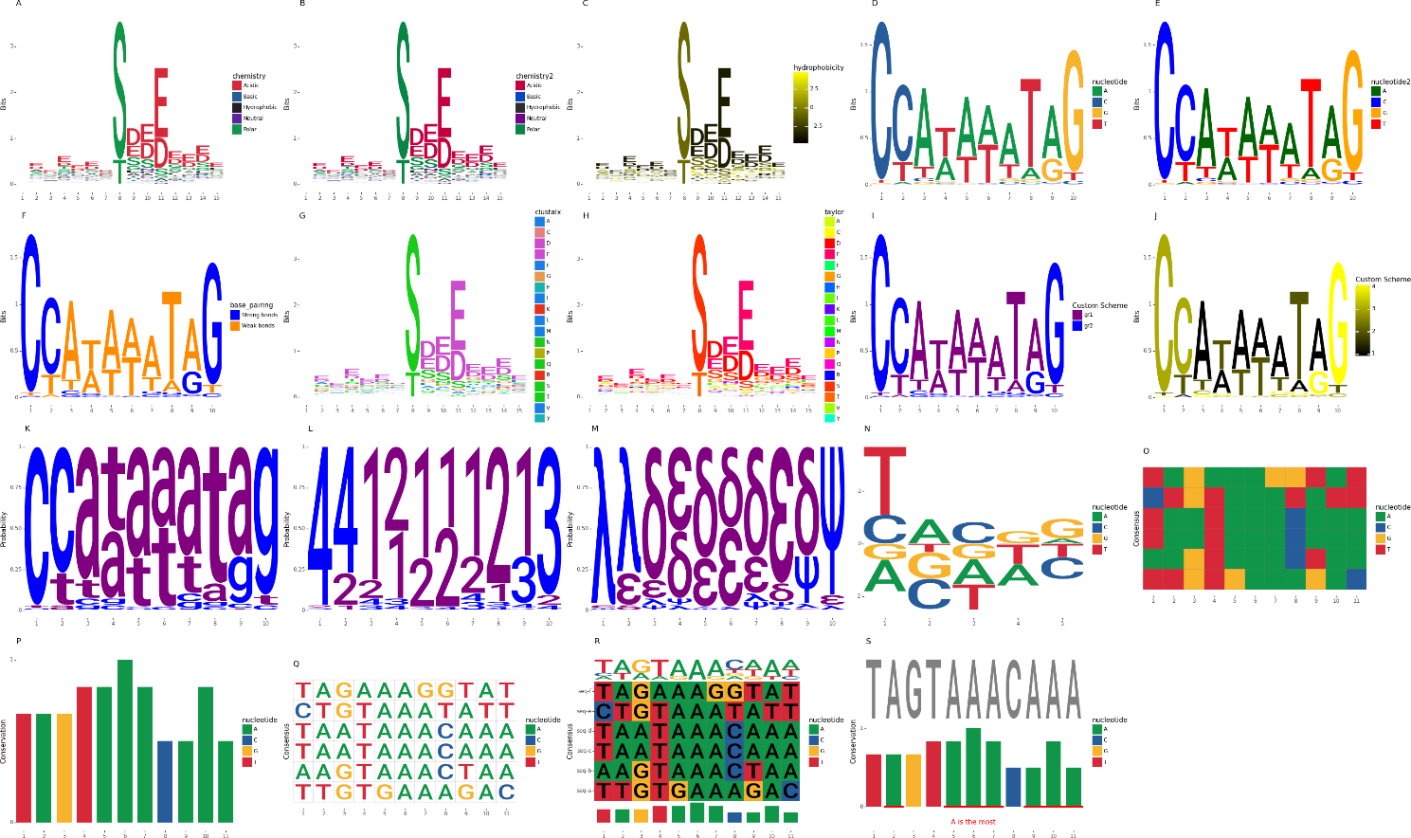
Use plotnineSeqSuite to draw various graphs. **A**) draw a sequence logo based on the ‘chemistry’ color scheme, **B**) draw a sequence logo based on the ‘chemistry2’ color scheme, **C**) draw a sequence logo based on the ‘hydrophobicity’ color scheme, **D**) draw a sequence logo based on the ‘nucleotide’ color scheme, **E**) draw a sequence logo based on the ‘nucleotide2’ color scheme, **F**) draw a sequence logo based on the ‘base_pairing’ color scheme, **G**) draw a sequence logo based on the ‘clustalx’ color scheme, **H**) draw a sequence logo based on the ‘taylor’ color scheme, **I**) custom discrete color scheme, **J**) custom continuous color scheme, **K**) use lowercase English letters to draw a sequence logo, **L**) use numeric characters to draw a sequence logo, **M**) use special symbols to draw a sequence logo, **N**) draw a custom height logo, **O**)draw a sequence alignment diagram in no letter mode, **P**) draw a sequence histogram in no letter mode, **Q**) draw a sequence alignment diagram in which color schemes are applied to characters, **R**) drawn all layers in the same coordinate system, **S**) use with other functions of plotnine

plotnineSeqSuite predefines 8 color schemes (Color schemes), including 3 nucleic acids and 5 amino acids, which can be applied to the color of the logo of geom_logo, the color of the cylinder of geom_seqBar and the color of the background square or character of geom_alignedSeq (Fig. 2A-H). In addition, plotnineSeqSuite defines make_col_scheme() function that be used to customize the color scheme very easily(Fig. 2I-J).

### Custom alphabet and custom height logos

plotnineSeqSuite can not only draw English letters representing amino acids and nucleic acids, but also supports letters of any upper and lower case, numbers, and special symbols (Fig. 2K-M). When calling the init() function of the layer class, the above functions can be realized by passing a custom alphabet to the parameter namespace. Conventional sequence logos only have two modes: probability and bits. For other unconventional sequence logos, plotnineSeqSuite can customize the height of logos. When calling the init() function of class geom_logo, users can set the parameter method to ‘custom’. At this time, the type of data that passes in must be NumPy.ndarray, but it does not have to be a PFM. geom_logo will draw letters according to the value in NumPy.ndarray, whose value can even be negative (Fig. 2N). Furthermore, geom_alignedSeq and geom_seqBar support no letter mode (Fig. 2O-P), and geom_alignedSeq supports modes in which color schemes are applied to characters (Fig. 2Q).

### Drawn in the same coordinate system

When studying the commonality and differences between multiple gene sequences, developers often need to display different types of graphs together. There are two solutions for this situation, one is to use collage software to combine multiple pictures into one, and the other is to draw these pictures in the same coordinate system. Because the design of the layers of plotnineSeqSuite are object-oriented, users can change the final presentation form of the layer by adjusting the value of the property of the corresponding layer class. After understanding the meaning of the DataFrame of each layer, the user can easily draw various pictures of plotnineSeqSuite in the same coordinate system (Fig. 2R).

### Compatibility with other functions of plotnine

As plotnineSeqSuite is an extension of plotnine which is the Python implementation version of ggplot2, users can use the functions of plotnineSeqSuite and ggplot2 at the same time without hindrance (Fig. 2S).

## Discussion

### Compatibility with similar APIs in R

**Fig. 3:**
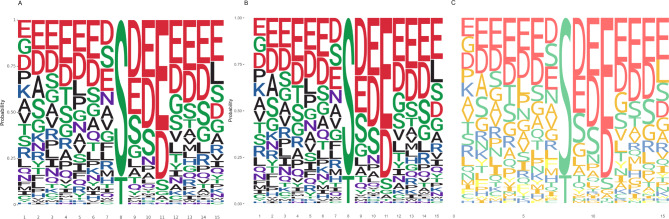
Logos generated by three software. **A**) use plotnineSeqSuite to draw a probability logo, **B**) use ggseqlogo to draw a probability logo, C) use ggmsa to draw a probability logo

This chapter will use plotnineSeqSuite and similar software packages in R to write code to draw a sequence logo. The R packages used in this chapter are ggseqlogo and ggmsa [41]. ggseqlogo has been mentioned in the background chapter. ggmsa is a recently published R package whose main function is to visualize multiple sequence alignments. At the same time, it can also draw sequence logos. We use plotnineSeqsuite, ggseqlogo and ggmsa to generate probability logos respectively (Fig. 3A-C). After read the code [see Additional file 3], it can be found that the code used by plotnineSeqsuite in Python is similar to that used by ggseqlogo and ggmsa in R. They can all be applied with ggplot2 functions by using the plus sign (+). For example, they both use the function ggtitle() to set the title and the function theme() to adjust the drawing style.

### Feature comparison

This section will compare the features of existing similar R packages and Python packages. ggseqlogo and ggmsa have been introduced above. Logomaker is a recently released Python package which provides a programming API for drawing sequence logos.

Table 2 shows the features comparison between plotnineSeqSuite, ggseqlogo, Logomaker and ggmsa. Unlike ggseqlogo and ggmsa, the programming paradigm of plotnineSeqSuite and Logomaker is object-oriented. Each graph is represented by a Python class. Users can implement secondary development by changing the properties of objects or inheriting classes.

**Table 2:** Features comparison between plotnineSeqSuite, ggseqlogo and ggmsa

		plotnineSeqSuite	Logomaker	ggseqlogo	ggmsa
Programming language		Python	Python	R	R
Framework library		plotnine	Matplotlib	ggplot2	ggplot2
Programming style		ggplot2	Matplotlib	ggplot2	ggplot2
Programming paradigm		Object-oriented	Object-oriented	Process-oriented	Process-oriented
Sequence type	DNA	Yes	Yes	Yes	Yes
	RNA	Yes	Yes	Yes	Yes
	AA	Yes	Yes	Yes	Yes
	Custom letters	Yes	Yes	Yes	Not supported
Sequence logo	Probability logo	Yes	Yes	Yes	Yes
	Information logo	Yes	Yes	Yes	Not supported
	Custom logo	Yes	Yes	Yes	Not supported
Sequence alignment diagram		Yes	Not supported	Not supported	Yes
Sequence histogram		Yes	Not supported	Not supported	Yes

The framework library that plotnineSeqSuite relies on is plotnine while Logomaker relies on Matplotlib [35]. The framework library that ggseqlogo and ggmsa rely on is ggplot2. The programming styles of plotnineSeqSuite, ggseqlogo and ggmsa are all ggplot2 style while Logomaker is Matplotlib style. In terms of programming style, ggplot2 is easier to use than Matplotlib [42].

In addition to supporting standard nucleic acid and amino acid sequences, plotnineSeqSuite, Logomaker and ggseqlogo also support sequences consisting of numbers, other English letters, and special symbols. Unfortunately, ggmsa can only plot sequence logos in probability mode. However, plotnineSeqSuite, Logomaker and ggseqlogo can plot sequence logos in bits mode and probability mode, and both of them can even customize the height of the letter by passing a position-height matrix. plotnineSeqSuite and ggmsa can plot sequence alignment diagrams, sequence logos and sequence histograms, while ggseqlogo and Logomaker focuses only on sequence logos.

### A case study

To introduce how plotnineSeqSuite works, we reproduce the work of Momont et al. as an example [43]. In their article, they used non-standard probability logos to show the results of data analysis. The original words are: “Logo plot amino acid conservation of SA, OSE, FNI9, FNI17, FNI19 and 1G01 epitopes based on available NA sequences from human seasonal H1N1 (n = 64,476) and H3N2 (n = 91,754) IAVs (h) and Victoria/ 2/87-like (n = 23,787) and Yamagata/16/88-like (n = 17,769) IBVs and Key contact residues are shown in red” [43].

Code [see Additional file 4] and instructions are below.

Step 1. The user needs to import the necessary modules of plotnine and plotnineSeqSuite, and simulate the generated data (the author does not publish the input data of the plot).

**Fig. 4:**
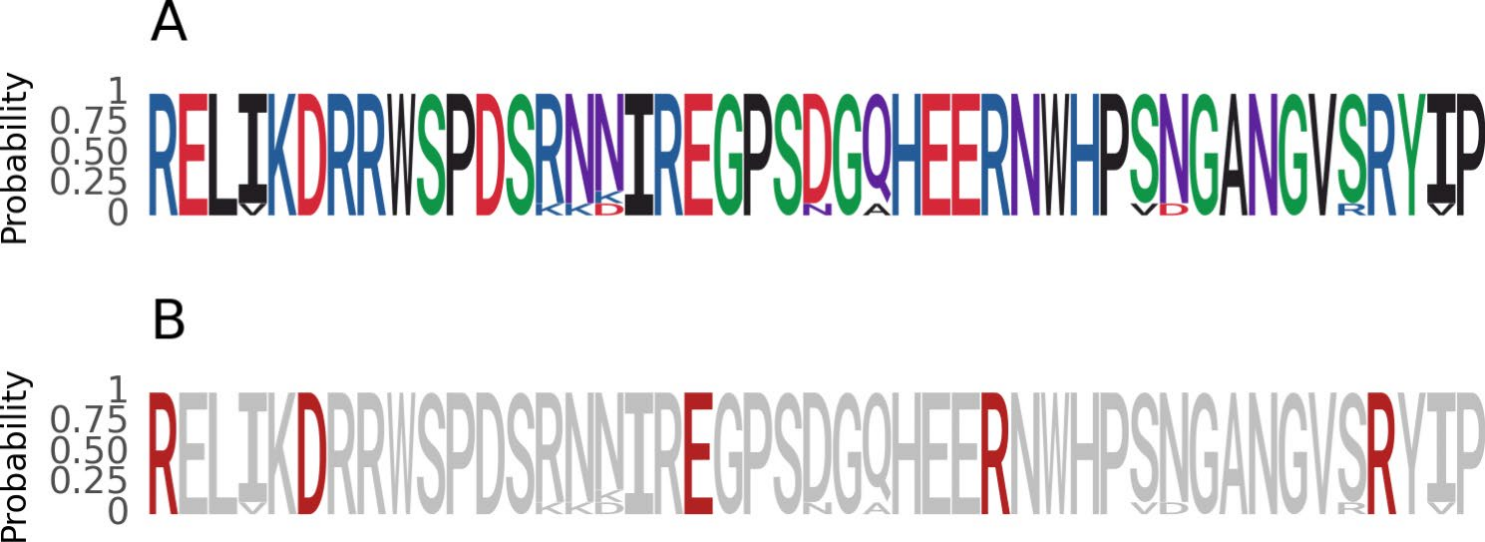
Reproduce the logo. **A**) the probability reproduced logo base the default color scheme, **B**) the reproduced probability logo based a special color scheme

Step 2. Users need to modify the color scheme. The logo does not determine the fill color based on the value of the letter (Fig. 4A) but based on the position. For example, the letter R is red in the first position and gray in the seventh position (Fig. 4B). By default, plotnineSeqSuite do not provide an API for configuring such a color scheme. But plotnineSeqSuite is object-oriented, and the data source of geom_logo drawing is one of its properties. geom_logo can change the default color scheme by adjusting the property value and using the function scale_fill_manual().

Step 3. The user uses the function theme() and guides() to adjust the style of the axis and generate a picture.

## Conclusions

plotnineSeqSuite provides an all-in-one tool for drawing graphs related to gene sequences, which is developed based on plotnine (Python version of ggplot2). As a consequence, users can easily get started and do various DIY based on ggplot2 functions. In other words, plotnineSeqSuite unifies the drawing of graphs related to gene sequences in R and Python. Since this package is based on object-oriented development, users can inherit and encapsulate it easily.

### Electronic supplementary material

Below is the link to the electronic supplementary material.

Supplementary Material 1

Supplementary Material 2

Supplementary Material 3

Supplementary Material 4

## Data Availability

Source code is available in https://pypi.org/project/plotnineseqsuite and https://github.com/caotianze/plotnineseqsuite. Documentation and tutorials can be found at https://caotianze.github.io/plotnineseqsuite/.

## Declarations

### Availability and requirements

Project name: plotnineSeqSuite.

Project home page: https://pypi.org/project/plotnineseqsuite.

Operating system(s): any supporting Python > = 3.10 (tested on Windows 10 and Ubuntu 20.04) and can run the pip command to install Python modules online.

Programming language: Python.

Other requirements: plotnine = = 0.12.2.

License: MIT License.

Any restrictions to use by non-academics: commercial use requires permission from the designer of the fonts.

Installation: open the command line program of your operating system. When your computer is connected to the Internet, you only need to enter “pip install plotnineSeqSuite” in the command line program, and the program will be downloaded and installed automatically.

## List of abbreviations

DNA: DeoxyriboNucleic Acid
API: Application Programming Interface
PFM: Position Frequency Matrix
DIY: Do It Yourself
