# Supplementary material for: plotnineSeqSuite: a Python package for visualizing sequence data using ggplot2 style
Source: BMC Genomics. 2023 Oct 3;24:585. doi: 10.1186/s12864-023-09677-8 (PMC10546746; doi:10.1186/s12864-023-09677-8)
Supplement: Supplementary file 1 — Supplementary Material 1 [file 12864_2023_9677_MOESM1_ESM.pdf]

Additional file 1.pdf

The formula for calculating the height of the information logo.

The information content (y-axis) of position i is given by:

for amino acids,

$$R_i = \log_2(20) - (H_i + e_n)$$

for nucleic acids,

$$R_i = \log_2(4) - (H_i + e_n)$$

where H_i is the uncertainty (sometimes called the Shannon entropy) of position i

$$H_i = -\sum_{b=1}^{t} f_{b,i} \times \log_2 f_{b,i}$$

Here, $f_{b,i}$ is the relative frequency of base or amino acid b at position i, and e_n is the small-sample correction for an alignment of n letters. The height of letter b in column i is given by

$$\text{height} = f_{b,i} \times R_i$$

The approximation for the small-sample correction, e_n, is given by:

$$e_n = \frac{1}{\ln 2} \times \frac{s-1}{2n}$$

where s is 4 for nucleotides, 20 for amino acids, and n is the number of sequences in the alignment.

References

Sequence logo. https://en.wikipedia.org/wiki/Sequence_logo. Accessed 08 September 2023
