# Supplementary material for: plotnineSeqSuite: a Python package for visualizing sequence data using ggplot2 style
Source: BMC Genomics. 2023 Oct 3;24:585. doi: 10.1186/s12864-023-09677-8 (PMC10546746; doi:10.1186/s12864-023-09677-8)
Supplement: Supplementary file 2 — Supplementary Material 2 [file 12864_2023_9677_MOESM2_ESM.pdf]

Additional file 2.pdf

Table S1-S4. Table S1: Columns of geom_alignedSeq.letter_data. Table S2: Columns of geom_seqBar.bar_data. Table S3: Columns of geom_seqBar.letter_data. Table S4: Columns of geom_logo.data.

Table S1: Columns of geom_alignedSeq.letter_data

Number	Name	Example	Function
1	letter	A	The column is used to identify which letter the row is used to draw.
2	position	6	The column is used to identify at which position the data is used to plot the aligned sequences.
3	y_index	0	The column is used to identify index of y-axis at current aligned position.
4	x	5.625	The column is used to map x in geom_polygon().
5	y	0.12641	The column is used to map y in geom_polygon().
6	order	1	The column is used to sort the data in geom_polygon().
7	seq_group	1	The column is used in facet_wrap().When the type of input data is dict, the value is

			the key value of dict. In other cases, the default is 1.
8	group_by	1.A.6.0	The column is used to map group in geom_polygon().
9	col	#D62839	When the color scheme is discrete, this column is used to specify the corresponding color. The column only exists when the parameter scheme_applied is 'LETTER' when the class geom_alignedSeq is constructed.
10	group	T	The column is used to map fill in geom_tile(). The column only exists when the parameter scheme_applied is 'LETTER' when the class geom_alignedSeq is constructed.

Table S2: Columns of geom_seqBar.bar_data

Number	Name	Example	Function
1	letter	T	The column is used to identify which letter the row is used to draw.
2	position	1	The column is used to identify at which position the data is used to plot the aligned sequences.
3	frequency	4.0	The column is used to determine how often letters appear in alignment positions.

4	x	1	The column is used to map x in geom_tile().
5	y	0.33	The column is used to map y in geom_tile().
6	width	0.75	The column is used to map width in geom_tile().
7	height	0.67	The column is used to map height in geom_tile().
8	seq_group	1	The column is used in facet_wrap().When the type of input data is dict, the value is the key value of dict. In other cases, the default is 1.
9	col	#D62839	When the color scheme is discrete, this column is used to specify the corresponding color.
10	group	T	The column is used to map fill in geom_tile().

Table S3: Columns of geom_seqBar.letter_data

Number	Name	Example	Function
1	letter	A	The column is used to identify which letter the row is used to draw.
2	position	2	The column is used to identify at which position the data is used to plot the aligned sequences.

3	order	1	The column is used to sort the data in geom_polygon().
4	x	1.625	The column is used to map x in geom_polygon().
5	y	1.12641	The column is used to map y in geom_polygon().
6	seq_group	1	The column is used in facet_wrap().When the type of input data is dict, the value is the key value of dict. In other cases, the default is 1.
7	group_by	1.A.2	The column is used to map group in geom_polygon().

Table S4: Columns of geom_logo.data

Number	Name	Example	Function
1	letter	A	The column is used to identify which letter the row is used to draw.
2	position	1	The column is used to identify at which position the data is used to plot the aligned sequences.
3	order	1	The column is used to sort the data in geom_polygon().
4	x	0.525	The column is used to map x in

			geom_polygon().
5	y	0.00431	The column is used to map y in geom_polygon().
6	seq_group	1	The column is used in facet_wrap().When the type of input data is dict, the value is the key value of dict. In other cases, the default is 1.
7	col	#109648	When the color scheme is discrete, this column is used to specify the corresponding color.
8	group	A	The column is used to map fill in geom_polygon().
9	group_by	1.A.1	The column is used to map group in geom_polygon().
