# Supplementary material for: plotnineSeqSuite: a Python package for visualizing sequence data using ggplot2 style
Source: BMC Genomics. 2023 Oct 3;24:585. doi: 10.1186/s12864-023-09677-8 (PMC10546746; doi:10.1186/s12864-023-09677-8)
Supplement: Supplementary file 3 — Supplementary Material 3 [file 12864_2023_9677_MOESM3_ESM.pdf]

Additional file 3.pdf

Code for generate Fig. 3A-B.

1. Python script used to generate Fig. 3A.png.

```
from plotnine import ggplot, ggtitle, theme, guides, element_blank, element_rect
from plotnineseqsuite.data import seqs_aa
from plotnineseqsuite.logo import geom_logo

A=ggplot() + geom_logo(seqs_aa['CSNK2A2'], method='probability',
col_scheme='chemistry') + ggtitle('A')+ guides(fill=False)
A=A+theme(panel_grid=element_blank(),panel_background=element_rect(fill='white'),a
xis_ticks_major_x=element_blank())

A.save("Fig. 3A.png",width=7,height=7,dpi=300)
```

2. R script used to generate Fig. 3B.png.

```
library(ggplot2)
library(ggseqlogo)
data(ggseqlogo_sample)

A=ggplot() + geom_logo(seqs_aa[['CSNK2A2']], method = 'probability',
col_scheme='chemistry')+ggtitle('B')+guides(fill=FALSE)
A=A+theme(panel.grid=element_blank(),panel.background=element_rect(fill='white'),a
xis.ticks.x=element_blank())

ggsave('Fig. 3B.png',width=7,height=7,dpi=300)
```

3. R script used to generate Fig. 3C.png.

```
library(ggseqlogo)
library(Biostrings)
data(ggseqlogo_sample)
library(ggplot2)
library(ggmsa)
aastr=seqs_aa[['CSNK2A2']]
names(aastr)=1:80
seqstr = AAStringSet(aastr)

A=seqlogo(seqstr, adaptive = TRUE)+ggtitle('C')
A=A+theme(panel.grid=element_blank(),panel.background=element_rect(fill='white',co
lor=NA),plot.background=element_rect(fill='white',color=NA),axis.ticks.x=element_b
```

```
lank())

ggsave('Fig. 3C.png',width=7,height=7,dpi=300)
```
