# Supplementary material for: plotnineSeqSuite: a Python package for visualizing sequence data using ggplot2 style
Source: BMC Genomics. 2023 Oct 3;24:585. doi: 10.1186/s12864-023-09677-8 (PMC10546746; doi:10.1186/s12864-023-09677-8)
Supplement: Supplementary file 4 — Supplementary Material 4 [file 12864_2023_9677_MOESM4_ESM.pdf]

Additional file 4.pdf

Code used to generate Fig. 4B.png

Step 1

```
from plotnine import ggplot, theme, guides, element_text, element_rect,
element_blank, scale_fill_manual
from plotnineseqsuite import geom_logo
data = ['RELVKDRRWSPDSKKKIREGPSNGAHEERNWHPVDGANGVRRYVP',
'RELIKDRRWSPDSRNDIREGPSDGQHEERNWHPSNGANGVSRYIP','RELIKDRRWSPDSRNNIREGPSDGQHEERNWHP
SNGANGVSRYIP',
'RELIKDRRWSPDSRNNIREGPSDGQHEERNWHPSNGANGVSRYIP','RELIKDRRWSPDSRNNIREGPSDGQHEERNWHP
SNGANGVSRYIP',
'RELIKDRRWSPDSRNNIREGPSDGQHEERNWHPSNGANGVSRYIP','RELIKDRRWSPDSRNNIREGPSDGQHEERNWHP
SNGANGVSRYIP',
'RELIKDRRWSPDSRNNIREGPSDGQHEERNWHPSNGANGVSRYIP','RELIKDRRWSPDSRNNIREGPSDGQHEERNWHP
SNGANGVSRYIP', 'RELIKDRRWSPDSRNNIREGPSDGQHEERNWHPSNGANGVSRYIP']
```

Step 2

```
layer_logo = geom_logo(data=data, method='probability')
def change_group(x):
    if x['position'] ==1 or x['position'] ==6 or x['position'] ==19 or
x['position'] ==29 or x['position'] ==42:
        x['group']='interested'
    else:
        x['group']='not_interested'
    return x
layer_logo.data = layer_logo.data.apply(func=change_group, axis=1)
g = ggplot() + layer_logo +
scale_fill_manual({'interested':'#B22222','not_interested':'#C0C0C0'})
```

Step 3

```
g = g + theme(aspect_ratio=0.1, panel_grid=element_blank(),
panel_background=element_rect(fill='white'),axis_title_x=element_blank(),
axis_text_x=element_blank(),axis_ticks_major_x=element_blank(),axis_ticks_major_y=
element_blank(),axis_title_y=element_text(size=8)) + guides(fill=False)
g.save('Fig. 4B.png',dpi=300)
```
